# The Gil Classification: A Novel Anatomical and Surgical Staging System for Reverse Abdominoplasty

**DOI:** 10.7759/cureus.115724

**Published:** 2026-09-03

**Authors:** Luis Atilio Gil Perez, Ahmed Qaraqra

**Affiliations:** 1 Plastic and Reconstructive Surgery, Hospital Real San Jose de Valle Real, Guadalajara, MEX

**Keywords:** body-contouring surgery, classification system, reverse abdominoplasty, reverse abdominoplasty flap, therapeutic abdominoplasty

## Abstract

Reverse abdominoplasty is a body-contouring and reconstructive technique in which redundant upper abdominal skin and soft tissue are resected through an inframammary incision and the resulting flap is advanced cephalad rather than caudally, as in conventional abdominoplasty. Although first described more than five decades ago, the procedure remains comparatively underused, and its literature is limited to small retrospective case series without a shared framework for grading the severity of upper abdominal laxity or standardizing operative planning.

The authors reviewed the published literature on reverse abdominoplasty spanning esthetic, post-bariatric, and reconstructive applications and used it, together with clinical experience, to develop a four-tier anatomical and surgical classification system, herein termed the Gil classification. Type I describes laxity confined to the upper hemiabdomen with a preserved myoaponeurotic wall, managed through bilateral crescentic incisions confined to the inframammary fold with conservative adjunctive liposuction. Type II reflects similar aponeurotic integrity but a thicker adipofascial flap that requires a continuous horizontal inframammary incision and more extensive liposuction to achieve flat coaptation. Type III introduces myoaponeurotic compromise localized to the upper hemiabdomen and periumbilical region, requiring supra- and, selectively, infraumbilical plication. Type IV denotes generalized myoaponeurotic weakness and diastasis affecting the entire abdominal wall, requiring an extended submammary incision, plication in both vertical and horizontal planes, and a T- or H-pattern approach. For each tier, expected skin laxity, aponeurotic status, liposuction strategy, incision pattern, plication requirements, and compatible synergistic procedures (mastopexy, augmentation, or reduction mammaplasty; oncologic or burn reconstruction) were defined. This was accompanied by tier-specific technical considerations, including strategies to prevent the compensatory “pouching effect” after superior plication and principles for preserving perforator-based flap vascularity. This classification is intended to standardize preoperative assessment, guide operative planning, and provide a common reference for future comparative studies, addressing a gap that the existing reverse abdominoplasty literature has not previously addressed.

## Introduction

Reverse abdominoplasty is a body-contouring and reconstructive procedure in which excess upper abdominal skin and subcutaneous tissue are resected through an inframammary incision, with the resulting flap advanced cephalad toward the chest rather than caudally as in conventional abdominoplasty. The technique was first popularized as an advancement flap for correcting upper abdominal wall deformities and was subsequently combined with reduction mammaplasty since both procedures can share the same inframammary scar [1]. Later refinements in the understanding of the perforator-based vascular supply of the anterior abdominal wall broadened the range of patients for whom the flap could be safely raised [2].

Since these early descriptions, reverse abdominoplasty has evolved from a niche aesthetic maneuver into a versatile tool spanning post-bariatric body contouring, autologous breast augmentation, and oncologic and burn reconstruction. Agha-Mohammadi and Hurwitz reported the largest published series, describing 88 reverse abdominoplasties performed within total-body-lift contours after massive weight loss, with a 5% complication rate [3]. Zienowicz and Karacaoglu described augmentation mammaplasty using de-epithelialized upper abdominal adipofascial flaps (AMBRA) in 37 patients [4]. In the reconstructive domain, the same inframammary approach has been used to close mastectomy defects in locally advanced breast cancer [5,6], manage oncologic trunk defects [7], salvage infected implant-based breast reconstructions [8], and correct epigastric skin retraction after burn injury [9].

Despite this expanding range of indications, the literature on reverse abdominoplasty remains sparse relative to conventional abdominoplasty and consists almost exclusively of small retrospective case series and case reports, without randomized or comparative studies [7,10]. Halbesma and van der Lei’s seven-case series and accompanying English-language literature review remain among the most frequently cited sources on the topic [10]. Reported outcomes are generally favorable, with major complication rates below 10% across most series [3,11], but complication reporting is inconsistent across studies and validated patient-reported outcome measures are rarely used [12].

A recurring limitation of this body of work is the absence of any classification system that stratifies patients by the anatomical severity of upper abdominal laxity, myoaponeurotic wall integrity, and the extent of the required surgical maneuver. Existing reports instead describe individualized approaches to specific scenarios, for example, patients with pre-existing bi-subcostal scars [13], staged combination with mini-abdominoplasty [14], or extended dissection to the pubis for single-scar contouring of the entire abdominal wall [15], but to the authors' knowledge, no dedicated framework currently exists to guide preoperative planning, standardize technique selection, or permit comparison of outcomes across centers or studies. In this technical report, we introduce a four-tier anatomical and surgical classification system, the Gil classification, proposed to address this gap.

## Technical report

Development of the classification

The Gil classification was developed by the two authors through a non-systematic (narrative) review of the published aesthetic and reconstructive reverse abdominoplasty literature identified via PubMed, combined with their own clinical experience performing the procedure. No structured or systematic search protocol, formal consensus methodology (e.g., Delphi process or nominal group technique), or external expert panel was used at this stage, and this is acknowledged as a limitation throughout this report; the classification should accordingly be regarded as a preliminary, literature- and experience-derived conceptual framework rather than an evidence-graded or consensus-validated staging system.

The four classification axes were not predetermined; they were derived inductively after a review of the available literature and the authors' cases, by identifying which preoperative and intraoperative variables recurred most consistently across published series as determinants of operative extent. These are: (i) the distribution and severity of skin laxity; (ii) the integrity of the myoaponeurotic wall, principally the presence and extent of rectus diastasis; (iii) the extent and pattern of adjunctive liposuction required to thin the flap; and (iv) the incision pattern needed to resect and redrape the flap, together with the propensity for rectus plication and for combining the procedure with a synergistic breast operation. These four axes were used to stratify patients into four sequential tiers of increasing anatomical and surgical complexity, summarized in Table 1.

**Table 1:** Schematic progression of the Gil classification (Types I-IV)

Type	Skin Laxity	Myoaponeurotic Wall	Adjunctive Liposuction	Incision/Approach	Rectus Plication	Synergistic Procedures	Key Surgical Considerations
I	Mild, confined to the upper hemiabdomen.	Preserved; no clinically evident diastasis or laxity.	Localized and conservative, oriented toward micro-definition of the contour.	Bilateral crescentic incisions strictly confined to the inframammary fold.	Not indicated.	Mastopexy or augmentation mammaplasty in the same operative setting.	Mammary integration: the reduced incision allows a clean dual approach without compromising breast vascularity or altering the inframammary fold position.
II	Moderate, circumscribed to the upper hemiabdomen.	Preserved; profile deformity is secondary to adipose panniculus volume.	Directed at the upper abdominal flap for subcutaneous lipodystrophy.	Continuous horizontal incision along the bilateral inframammary fold.	Not indicated.	Reductive or reconstructive mastopexy/mammaplasty.	Flap-thickness management: bulging is purely tissue-related; requires panniculus thinning by liposuction for precise, tension-free flap coaptation.
III	Severe, involving the upper hemiabdomen and periumbilical region.	Focalized structural compromise: rectus diastasis and laxity of the superior aponeurosis.	Extensive, in the superior flap, to optimize thinning and define the thoraco-abdominal transition.	Continuous horizontal incision along the bilateral inframammary fold.	Supra-umbilical plication; selective infraumbilical plication by mini-incision or endoscopic approach per intraoperative findings.	Mastopexy; correction of residual aponeurotic deformities of the lower hemiabdomen.	Prevention of the compensatory “pouching effect”: superior plication tends to displace tissue mass and increase infraumbilical laxity; infraumbilical intervention is decided by dynamic intraoperative assessment.
IV	Severe and global; affects the entire abdominal wall (supra- and infraumbilical).	Generalized myoaponeurotic weakness and severe diastasis throughout the abdominal wall.	Deep, selective, and controlled.	Complete submammary incision with lateral extensions, or vertical midline incision (T- or H-pattern).	Extended supra- and infraumbilical myoaponeurotic plication in both vertical and horizontal planes.	Complex reconstruction of body contour; combined breast surgery contingent on hemodynamic stability and systemic status.	Vascular and biomechanical dynamics: liposelection must prioritize preservation of epigastric and intercostal perforators for flap viability; the broad T/H incision pattern accommodates bidirectional perforator recruitment.

Two of these axes--skin laxity and myoaponeurotic wall integrity--are anatomical, patient-side descriptors intended to characterize the deformity itself; the remaining axes--liposuction strategy, incision pattern, plication, and synergistic procedures--describe an associated operative strategy that the authors currently favor for each anatomical tier. This report does not treat the two as strictly separable; in the authors' practice, the anatomical findings and the operative response to them are assessed together, and the classification is intended to function simultaneously as a descriptive anatomical stage and as a starting operative reference. However, the operative components should not be read as a mandatory algorithm. Actual technique selection is also influenced by factors the classification does not capture, including body mass index, tissue quality, prior abdominal or breast surgery and scarring, comorbidities, and whether breast surgery is performed concurrently; surgeons should adapt the suggested approach to individual patient findings.

In its current form, all four axes are assessed by standard clinical examination: inspection and manual palpation of skin laxity and rectus diastasis in the standing and supine positions, with the standard pinch and rectus-separation maneuvers used in conventional abdominoplasty assessment. Imaging (ultrasound or, where already available for other indications, CT) may be used adjunctively to confirm diastasis width but is not required. The descriptors used to separate tiers--“mild,” “moderate,” “severe,” “focalized,” and “generalized”--are therefore qualitative and rely on clinical judgment; quantitative thresholds (for example, a defined inter-recti distance in centimeters separating Type I/II from Type III, or a defined pinch-test measurement separating mild from moderate skin laxity) have not yet been established and are not proposed here. Developing and validating such objective, reproducible criteria, together with formal inter-rater and intra-rater reliability testing, is identified as a priority for future work and is discussed further below.

Type I

Type I describes the mildest anatomical presentation: skin laxity confined to the upper hemiabdomen with a preserved myoaponeurotic wall and no clinically evident diastasis. This tier corresponds closely to the population described by Pacifico et al. and by Halbesma and van der Lei: typically, an older patient with excess upper abdominal skin, an existing inframammary scar from a prior conventional abdominoplasty, and no significant musculofascial deformity [10,11]. Because the aponeurosis is intact, the procedure is confined to bilateral crescentic incisions strictly within the inframammary fold with conservative, contour-oriented liposuction; rectus plication is not indicated. This tier is well suited to combination with mastopexy or augmentation mammaplasty through the shared incision, mirroring the dual aesthetic-reconstructive rationale of the AMBRA technique [4] and of combined series, such as Brunelli et al.’s, in which concurrent mammoplasty and reverse abdominoplasty shared a single inframammary scar [16].

Type II

Type II retains a preserved myoaponeurotic wall, but the profile deformity is driven by the volume of the adipose panniculus rather than skin excess alone. The flap is thicker, and the incision extends into a continuous horizontal line along the bilateral inframammary fold rather than a crescentic excision. Adjunctive liposuction is more extensive and is directed at thinning the superior flap, consistent with Ganandran et al.’s description of the perforator network, based on the deep and superficial inferior epigastric, superficial circumflex iliac, and lumbar vessels, which permits safe, liposuction-assisted thinning without compromising flap viability [2]. Because thickness rather than laxity drives this tier, achieving a flat, tension-free flap coaptation is the principal technical goal, an emphasis reflected in post-bariatric series, such as Allam et al.’s, in which inadequately thinned flaps were associated with seroma and wound-healing complications [17]. This tier is likewise compatible with concurrent reductive or reconstructive mammaplasty, as illustrated by Saldanha Filho et al.’s use of reverse mini-abdominoplasty flaps in implant-based breast reconstruction [18].

Type III

Type III introduces true myoaponeurotic compromise, focalized to the upper hemiabdomen and periumbilical region, with rectus diastasis and laxity of the superior aponeurosis. Liposuction becomes extensive across the superior flap to optimize thinning and define the thoraco-abdominal transition, and supra-umbilical rectus plication is required; infraumbilical plication is added selectively, through a mini-incision or endoscopic approach, according to intraoperative findings. The broader principle of extending dissection and plication to address both upper and lower laxity is supported by the extended single-scar technique described by Yacoub [15] and the staged combination of reverse and mini-abdominoplasty described by Yang et al. [14]; however, the specific choice between a mini-incision and an endoscopic approach for selective infraumbilical plication reflects the authors' current operative practice rather than a direct comparison reported in either source. Type III is also the tier at which synergistic procedures shift from purely aesthetic mammaplasty toward reconstructive indications, consistent with series combining chest-wall oncoplastic reconstruction with correction of residual aponeurotic deformity of the lower hemiabdomen [6,19].

A key technical consideration introduced at this tier is prevention of a phenomenon the authors term the compensatory “pouching effect”: because supra-umbilical plication tightens the upper aponeurosis, it appears, in the authors' clinical observation, to displace tissue mass and increase apparent laxity in the infraumbilical region. To the authors' knowledge, this specific compensatory pattern has not previously been named or characterized in the reverse abdominoplasty literature, and it is presented here as a proposed clinical observation rather than an established biomechanical phenomenon; it has not been quantified or studied prospectively. The decision to extend plication or resection below the umbilicus is therefore made intraoperatively, based on dynamic assessment of the abdominal wall after the superior plication is completed, rather than being fixed preoperatively.

Type IV

Type IV represents the most severe presentation: generalized myoaponeurotic weakness and diastasis affecting the entire abdominal wall, both supra- and infraumbilically. The incision is extended to a complete submammary line with lateral extensions, or converted to a vertical midline incision producing a T- or H-pattern, and plication is extended along both the vertical and horizontal planes. The scale of intervention at this tier parallels the total-body-lift context in which Agha-Mohammadi and Hurwitz performed their largest reported series [3] and the bi-subcostal scar technique described by Huttin et al. for extensive supra-umbilical excess [13]; the specific T- or H-pattern incision design and the combination of vertical and horizontal plication, however, are the authors' preferred technical solution for this degree of laxity rather than a technique directly validated by these sources. Because flap length and undermining are maximal at this tier, liposuction must be selective and controlled, prioritizing preservation of the epigastric and intercostal perforator vessels that Ganandran et al. identified as the dominant blood supply to the flap [2]; the broad T/H incision pattern is designed to accommodate this bidirectional perforator recruitment. Synergistic breast reconstruction remains possible at this tier but is contingent on the patient’s hemodynamic stability and overall systemic status given the magnitude of the combined procedure.

## Discussion

The Gil classification organizes a heterogeneous and largely case-based literature into a structure that mirrors how reverse abdominoplasty is planned in the authors' practice: by escalating the anatomical severity of skin laxity and myoaponeurotic compromise, and by the corresponding escalation of incision length, liposuction extent, and plication requirement. The published literature supporting upper abdominal laxity correction, autologous breast augmentation via the AMBRA technique, and mastectomy-defect closure consists of multiple broadly consistent, but uncontrolled and non-randomized, case series ranging from 6 to 88 patients [3-5,7,10,11]. No formal evidence-grading framework (such as GRADE) was applied to this body of literature, and in the absence of comparative or controlled data, this evidence base is best characterized as consistent but preliminary rather than assigned a formal grade of certainty. None of these series, moreover, stratifies patients by anatomical severity, which limits their comparability and makes it difficult to determine which technical modification is responsible for a given outcome. By defining explicit anatomical tiers, the classification is intended to make this kind of stratification possible in future work, though this has not yet been demonstrated.

Several recurring themes from the literature support the classification’s underlying axes. First, the procedure is repeatedly described as technically approachable, with a short learning curve for surgeons already familiar with conventional abdominoplasty and with adjuncts such as progressive tension sutures used to maintain flap position and limit scar migration [20]. Second, blood supply is consistently described as robust and perforator-based, which is what permits the liposuction-assisted flap thinning central to Types II through IV [2]. Third, the technique has repeatedly been shown to be safe even in patients with pre-existing subcostal or inframammary scars, supporting its use across the reoperative and post-bariatric populations that populate the lower tiers of the classification [1,13].

Limitations

This report has important limitations. The Gil classification was derived from a non-systematic synthesis of the published literature and the two authors' clinical experience rather than from a prospective, multicenter, or consensus-based process, and no patient cohort has yet been classified according to this system. As a direct consequence, several aspects of the classification's performance are currently unknown rather than merely unquantified: it is not known whether two independent surgeons examining the same patient would assign the same Gil type (inter-rater reliability), whether the same surgeon would assign the same type on repeated assessment (intra-rater reliability), or whether classification tier predicts operative complexity, complication rate, revision rate, operative duration, or patient-reported outcomes. Statements elsewhere in this report describing the classification as intended to standardize preoperative assessment or guide operative planning should accordingly be read as describing its intended future role rather than a demonstrated present capability. Existing outcome data for reverse abdominoplasty more broadly, as summarized above, are themselves limited by small sample sizes, retrospective design, and inconsistent complication reporting, and none of the reviewed series used a validated patient-reported outcome instrument, such as BODY-Q or BREAST-Q, to quantify satisfaction--a gap also highlighted in a recent systematic review of revision abdominoplasty outcomes [12]. A prospective, multicenter validation study, assessing inter-rater and intra-rater reliability, establishing quantitative diagnostic thresholds for each axis, and correlating tier assignment with operative time, complication rate, revision rate, and validated patient-reported outcomes, is required before the Gil classification can be considered a validated staging system rather than a proposed conceptual framework.

## Conclusions

Reverse abdominoplasty is a versatile but underreported technique spanning esthetic upper abdominal contouring, post-bariatric body reshaping, autologous breast augmentation, and oncologic and burn reconstruction, yet the literature that supports it has lacked a shared framework for describing anatomical severity or guiding operative planning. The Gil classification is proposed to address this gap by organizing patients into four sequential tiers (Types I through IV), defined by skin laxity, myoaponeurotic wall integrity, liposuction strategy, incision pattern, plication requirements, and compatible synergistic procedures, and ordered by increasing anatomical and surgical complexity rather than by a severity label attached to the procedure itself. At present, this is a preliminary, literature- and experience-derived conceptual framework; its descriptors are qualitative, its reliability between observers is untested, and no outcomes have yet been correlated with classification tier. Making these anatomical and technical distinctions explicit is intended to lay groundwork that may help standardize preoperative assessment and operative planning and provide a common reference point for future comparative and outcomes-based research in reverse abdominoplasty, pending the prospective validation described above.

## References

[1] Alves AS, Martineau J, Dupuis A, Zuo K, Kalbermatten DF, Oranges CM (2023). Reverse abdominoplasty in a patient presenting with subcostal nephrectomy scar and transverse muscle incisional hernia. Plast Reconstr Surg Glob Open.

[2] Ganandran T, Redit R, Mat Johar F, Wan Sulaiman WA, Halim AS (2022). Reversed abdominoplasty in aggressive locally advanced breast cancer defect: a safe option for high recurrence risk patients. Cureus.

[3] Agha-Mohammadi S, Hurwitz DJ (2010). Management of upper abdominal laxity after massive weight loss: reverse abdominoplasty and inframammary fold reconstruction. Aesthetic Plast Surg.

[4] Zienowicz RJ, Karacaoglu E (2009). Augmentation mammaplasty by reverse abdominoplasty (AMBRA). Plast Reconstr Surg.

[5] Whalen K, Liu L, Rejano CJ, Mhaskar R, Khakpour N, Dayicioglu D (2023). Reverse abdominoplasty for mastectomy defect closure in advanced breast cancer. Ann Plast Surg.

[6] Culbert M, Shock L, Fabricius MM, Nelson N (2022). Reverse abdominoplasty for reconstruction following oncologic resection of extensive breast disease. Cureus.

[7] Pantelides NM, Mondal D, Wishart GC, Malata CM (2013). Reverse abdominoplasty: a practical option for oncological trunk reconstruction. Eplasty.

[8] Mayer HF, Palacios Huatuco RM, Ramírez MF (2024). Reverse abdominoplasty as a salvage procedure for infected bilateral implant-based breast reconstruction. Int J Surg Case Rep.

[9] González YG, De Anda R, Soriano KG (2025). Inverted abdominoplasty as a reconstructive option in a patient with lipolaser burn sequelae. Int J Res Med Sci.

[10] Halbesma GJ, van der Lei B (2008). The reverse abdominoplasty. A report of seven cases and a review of English-language literature. Ann Plast Surg.

[11] Pacifico MD, Mahendru S, Teixeira RP, Southwick G, Ritz M (2010). Refining trunk contouring with reverse abdominoplasty. Aesthet Surg J.

[12] Nguyen AT, Bajaj K, Adam TH, Galiano RD (2025). Outcomes and indications in revision abdominoplasty: a systematic review. Ann Plast Surg.

[13] Huttin C, Hendriks S, Bodin F, Bruant-Rodier C, Ruffenach L, Dissaux C (2021). Reverse abdominoplasty in the presence of bi-subcostal scar: technical note [Article in French]. Ann Chir Plast Esthet.

[14] Yang X, Wang GH, Wang J, Xie HB (2018). Correction of contour deformity using reverse abdominoplasty combined with mini-abdominoplasty. Chin Med J (Engl).

[15] Yacoub CD (2016). Extended reverse abdominoplasty: a technical alternative for breast reconstruction. New Concepts on Abdominoplasty and Further Applications.

[16] Brunelli D, Mazzarella F, Zanettin C (2025). Reverse abdominoplasty: easily solving complicated situations. Case Reports Plast Surg Hand Surg.

[17] Allam A, Mostafa W, Hindy A, Allam A (2013). Reversed abdominoplasty for correction of abdominal contour deformities with supraumbilical scar post bariatric surgery. Egypt J Plast Reconstr Surg.

[18] Filho ORS, Saldanha O, Cação E (2017). Breast reconstruction with reverse mini abdominoplasty. Rev Bras Cir.

[19] Teklebrhan F, Mahir G, Clark S, Shanthakumar D, Patten DK, Ullah MZ (2021). Reverse abdominoplasty: a novel practical approach using oncoplastic reconstruction in managing major chest wall defects for patients with loco-regional recurrence following breast cancer surgery. Cureus.

[20] Deos MF, Arnt RA, Gus EI (2009). Tensioned reverse abdominoplasty. Plast Reconstr Surg.

